# WERFE: A Gene Selection Algorithm Based on Recursive Feature Elimination and Ensemble Strategy

**DOI:** 10.3389/fbioe.2020.00496

**Published:** 2020-05-28

**Authors:** Qi Chen, Zhaopeng Meng, Ran Su

**Affiliations:** ^1^School of Computer Software, College of Intelligence and Computing, Tianjin University, Tianjin, China; ^2^Military Transportation Command Department, Army Military Transportation University, Tianjin, China; ^3^Tianjin University of Traditional Chinese Medicine, Tianjin, China; ^4^Fujian Provincial Key Laboratory of Information Processing and Intelligent Control, Minjiang University, Fuzhou, China

**Keywords:** WERFE, gene selection, RFE, ensemble, wrapper

## Abstract

Gene selection algorithm in micro-array data classification problem finds a small set of genes which are most informative and distinctive. A well-performed gene selection algorithm should pick a set of genes that achieve high performance and the size of this gene set should be as small as possible. Many of the existing gene selection algorithms suffer from either low performance or large size. In this study, we propose a wrapper gene selection approach, named WERFE, within a recursive feature elimination (RFE) framework to make the classification more efficient. This WERFE employs an ensemble strategy, takes advantages of a variety of gene selection methods and assembles the top selected genes in each approach as the final gene subset. By integrating multiple gene selection algorithms, the optimal gene subset is determined through prioritizing the more important genes selected by each gene selection method and a more discriminative and compact gene subset can be selected. Experimental results show that the proposed method can achieve state-of-the-art performance.

## 1. Introduction

Gene expression data contains gene activity information, and it reflects the current physiological state of the cell, for example, whether the drug is effective on the cell, etc. It plays important roles in clinical diagnosis and drug efficacy judgment, such as assisting diagnosis and revealing disease occurrence mechanism (Lambrou et al., 2019). Gene expression data is rather complex, large in volume and grows fast. Since the dimensionality of gene expression data is often up to tens of thousands, it often consumes huge amount of time for analysis and it is difficult to make full use of it. The performance is not satisfied without proper processing. Although the dimensionality of gene expression data is extremely high, sometimes only a handful of the genes are informative and discriminative. Therefore, before the analysis of gene expression data, gene selection, which aims to reduce the dimensionality, is always carried out as the first step.

Gene selection is one special type of feature selection algorithm. It is a method to find the optimal gene subset from the original data set according to the actual needs (Su et al., 2019c). Over the years, many have studied the feature selection from different aspects. Kira et al. proposed a relief algorithm and defined the feature selection as a way to find the minimum feature subset that is necessary and sufficient to identify the target in ideal situations (Kira and Rendell, 1992). From the perspective of improving prediction accuracy, John et al. viewed the feature selection as a calculation procedure, which could increase classification accuracy or reduce the feature dimension without reducing the classification accuracy (John et al., 1994). In the definition of Koller et al.'s study, feature selection aims to select the smallest feature subset, and ensure that the predicted class distribution is similar to the original data class distribution (Koller and Sahami, 1996). In Dash et al.'s study, they considered the feature selection as a method to select a feature subset as small as possible, and meet conditions that not reduce the classification accuracy significantly and not change the class distribution significantly (Dash and Liu, 1997). Although the definition varied from study to study, they had the same goal, that is, to find a smallest feature subset to identify the target effectively and achieve an accuracy as high as possible. Their definition of feature selection takes into account both classification accuracy and class distribution. Based on algorithm model structure, feature selection method has been divided into three categories: filter, wrapper, and embedded method. The gene selection can also be divided into these three categories.

Filter method is an early feature selection method, which selects the optimal feature subset at the first place and then using this feature subset to train the model. The two steps are independent. Another way to think about it is that it measures the importance of each feature, ranks the features, selects the top ranked features, or the top ranked percentage of all the features as the final feature subset. This method has often been used to pre-process the raw data. Phuong et al. (2005) proposed an effective method filter-based method for finding tagging SNPs. In the study of Zhang et al.'s, the filter method is used to pre-process 3D image data (Zhang et al., 2015). Roffo et al. (2016) proposed a new filter-based feature selection method which achieved state-of-the-art performance.

Unlike filter method, wrapper method uses the output of the learning model as the evaluation criterion of each feature subset. In wrapper method, feature selection algorithm plays as an integral part of the learning algorithm, and the classification output is used to evaluate the importance of the feature subsets (here we focus on classification issues). By generating different combinations of genes, evaluating each combination, and then comparing between combinations, this type of approach eventually becomes an optimization problem in terms of determination of the finally selected subset. The wrapper algorithm has been studied extensively. Zhang et al. (2014) built a spam detection model and used a wrapper-based feature selection method to extract crucial features. Li Yeh et al. used the idea of wrapper algorithm, combined the tabu search and binary particle swarm optimization for feature selection, and successfully classified the micro-array data (Li Yeh et al., 2009). Shah et al. developed a new approach for predicting drug effect, and decision-tree based wrapper method was used in a global searching mechanism to select significant genes (Shah and Kusiak, 2004).

Wrapper method integrates feature selection process and model training process into one entirety (Su et al., 2019b). That is, the feature selection is carried out automatically during the learning process. This method is often coupled with well-performed classification methods such as support vector machine (SVM) or random forests (RF) in order to improve the classification accuracy and efficiency. Wrapper method has shown impressive performance in gene studies. Su et al. proposed a MinE-RFE gene selection method which conducted the gene selection inside the RF classification algorithm and achieved good performance (Su et al., 2019b). They also proposed a gene selection algorithm combing GeneRank and gene importance to select gene signatures for Non-small cell lung cancer subtype classification (Su et al., 2019f). The third class, embedded method, is similar to wrapper methods. Different from the wrapper method, an intrinsic model building metric is used during learning in embedded approach. Duval et al. (2009) presented a memetic algorithm which was an embedded approach dealing with gene selection for supervised classification of micro-array data. Hernandez and Hao (2007) tried a genetic embedded approach which performed the selection task combining a SVM classifier and it gave highly competitive results.

Ensemble strategy has been used widely to deal with diverse types of issues (Wei et al., 2017a,b, 2018a; Wang et al., 2018; Zhang W. et al., 2018; Su et al., 2019d; Zhang et al., 2019a). It takes advantages of different algorithms and the optimal outcome is obtained based on the optimization of the multiple algorithms. In this study, we propose an wrapper approach for gene selection, named WERFE, to deal with classification issues within a recursive feature elimination (RFE) framework. This WERFE employs an ensemble strategy, takes advantages of a variety of gene selection methods and assembles the top selected genes in each approach as the final gene subset. By integrating multiple gene selection algorithms, the optimal gene subset is determined through prioritizing the more important genes of each gene selection method. A more compact and discriminative gene subset is then selected.

## 2. Methodology

### 2.1. Data Sets and Preprocessing

In our study, we used five data sets to validate the proposed method, RatinvitroH, Nki70, ZQ_188D, Prostate and Regicor. RatinvitroH was retrieved from Open TG-GATEs database, which is a large-scale toxicogenomics database (https://toxico.nibiohn.go.jp/english/index.html). It stores gene expression profiles and toxicological data derived from *in* *vivo* (rat) and *in* *vitro* (primary rat hepatocytes and primary human hepatocytes) exposed to 170 compounds at multiple dosages and time points (Yoshinobu et al., 2015; Su et al., 2018). Here we identified hepatotoxic compounds based on the toxicogenomics data. We used the liver toxicogenomics data of rat *in* *vitro* and we selected the data at 24 h as at this time point the gene expression is higher in the single-dose study (Otava et al., 2014; Su et al., 2019e). All 31,042 genes of 116 compounds in the database were picked to build and estimate the gene selection method. Gene expression levels at three concentrations, low, middle, and high were recorded and we employed the response at the high concentration to represent the potency of the drugs. The gene expression was profiled with Affymetrix GeneChip.

Nki70 is a data set assembling expression of 70 breast cancer-related genes of 144 samples. CPPsite (http://crdd.osdd.net/raghava/cppsite/) is a manually curated database of experimentally validated 843 cell-penetrating peptides (CPPs) (Gautam et al., 2012), and CPPsite3.0 is the updated version of CPPsite2.0 (Piyush et al., 2015). ZQ_188D is derived from CPPsite3.0. It picks 188 CPPs of 9,024 samples. The Prostate data set contained 100 genes and 50 samples and it was used for cancer classification based on gene expression (Torrente et al., 2013). Regicor data set contained 22 genes and 300 samples (Subirana et al., 2014). It was used to identify death using cardiovascular risk factors. Table 1 shows the details of the five data sets we used in this study.

**Table 1:** The details of the five data sets.

**Dataset**	**Gene number**	**Sample number**
RatinvitroH	31,042	116
Nki70	70	144
ZQ_188D	188	9,024
Prostate	100	50
Regicor	22	300

#### 2.1.1. Support Vector Machine (SVM)

SVM is a widely used classification and regression analysis method in machine learning. It maps the raw data into high dimensional space through kernel functions to make the data linearly separable (Wang et al., 2019; Wei et al., 2019a,b). It was developed in Vapnik et al.'s study of statistical learning theory (Cortes and Vapnik, 1995), with the core idea to find the hyperplane between different categories, so that samples in different categories can be grouped into different sides of the separating hyperplane as far as possible. The early SVM was flat and limited. Then using more complicated kernel function, the application scope of SVM was greatly enlarged (Zhang N. et al., 2018).

SVM has the cost function as follows (Su et al., 2019a):

(1) $$\begin{array}{l} J\left(\theta \right)=C\overset{M}{\underset{i=1}{\sum }}\left[y^{i}cost_{1}\left(\theta^{T}x^{i}\right)+\left(1-y^{i}\right)cost_{0}\left(\theta^{T}x^{i}\right)\right]+\frac{1}{2}\overset{\gamma }{\underset{j=1}{\sum }}{\theta_{j}}^{2} \end{array}$$

where θ is the adjustable parameter of the model and γ is the number of θ; *M* is the number of the samples. *y*^*i*^ represents the category of the *i*-th sample. Here we considered binary classification with label 0 and 1. *cost*_1_ and *cost*_0_ are the objective function when *y*^*i*^ is equal to 1 and 0, respectively. *C* is the degree of penalty for controlling mis-classified training samples. It can only be set as a positive value. Here we used the SVM with linear kernel.

#### 2.1.2. Random Forest (RF)

Random forest (RF) is another classifier we used to train the model and obtain the importance of genes. RF is a method of discriminating and classifying data through voting of different classification trees (Ho, 1995; Gong et al., 2019; Lv et al., 2019). It is an ensemble learning method composed of multiple tree classifiers. It takes a random sample from the sample set with replacement, and then the samples are fed into the tree classifiers. Finally the class of the sample is determined by voting with the principle of majority rule. As it classifies the data, it can also provide the importance score of each variable (gene) and evaluate the role of each variable in the classification. In the process of applying RF, two parameters need to be determined. One is the number of samples selected each time and the other one is the number of decision trees in the random forest. The two parameters are determined according to the size of the data set.

### 2.2. Gene Selection Based on Recursive Feature Elimination

Gene selection was widely used in a number of fields (Fajila, 2019; Shahjaman et al., 2019). The most popular methods include Fisher-based methods (Gu et al., 2011), Relief-based methods (Robnik-Sikonja and Kononenko, 1997), FSNM methods (Nie et al., 2010), and mRMR (Peng et al., 2005) etc. All of these methods firstly rank the genes based on an evaluation criteria. Then based on the rank of genes, an appropriate gene subset is determined. However, the relationship between the number of selected genes and the classification precision cannot be fully reflected using these gene selection methods. Recently, Su et al. developed an algorithm balancing performance and gene number under the framework of recursive feature elimination (RFE) (Su et al., 2019b). Inspirited by their work, we designed the WERFE inside the RFE framework.

The RFE is a greedy algorithm which iteratively builds gene sets and the optimal subset is chosen from them. It was proposed by Guyon et al. with the intention to detect cancer (Guyon et al., 2002). The RFE iteratively eliminates the least important genes and conducts classification based on the new gene subsets. All the gene subsets are evaluated based on their classification performance. In our study, the finally selected subset is the one with the highest accuracy.

### 2.3. The Proposed Gene Selection Algorithm WERFE

#### 2.3.1. Gene Ranking Algorithm

**Figure 1:**
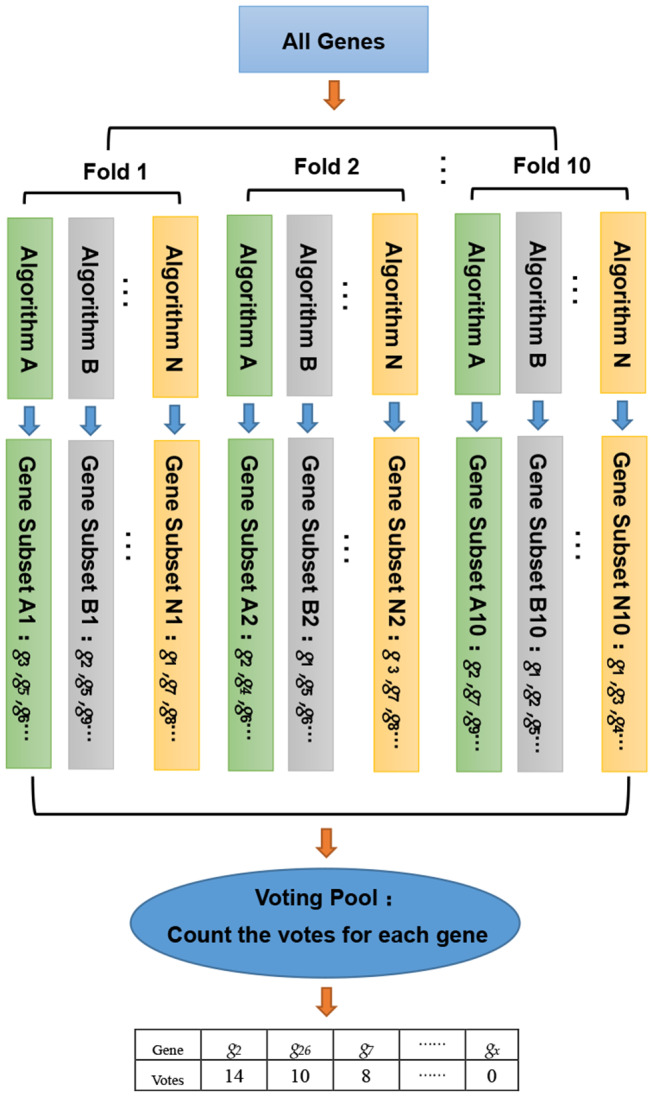
The entire process of gene ranking algorithm.

In this study, we developed a gene selection algorithm, named WERFE. Its main idea is to integrate two or more independent gene selection algorithms and the final decision is made based on all of these algorithms. The WERFE can be divided into two parts, the first is the gene ranking algorithm, and the second part is the determination of the optimal gene subset. Figure 1 illustrates the entire process of the gene ranking algorithm. Cross validation is widely used to evaluate the model (Liu et al., 2017; Zeng et al., 2017a, 2018). Therefore, the WERFE was performed inside a ten-fold cross validation procedure. In each fold, different gene selection algorithms used the training and test data to pick gene subsets. Then we put all the selected genes which were obtained from different algorithms into a voting pool (Chen et al., 2018). We counted the votes of each gene in the voting pool and ranked the genes based on the votes. In this way, we obtained a list of genes, *G*_*R*_, ranking from high to low. This ranking would be used for further gene selection. The pseudo code in Algorithm 1 shows the process of gene ranking. Here ten-Fold cross validation was used in WERFE, and two gene selection algorithms RF and SVM are integrated.

**Algorithm 1:** Gene ranking of Wrapper Embedded Recursive Feature Elimination (WERFE)

**Input**: Input data **X** : **x_1_**, **x_1_**...**x_m_** and labels **Y** : **y_1_**, **y_1_**...**y_m_**, where *m* is the number of samples. **x** is *n*-dimensional gene vector.*s* is the step size of RFE.
**Output**: Ranked genes *G*_*R*_ of all the genes.
1: **for** *k* = 1:10 **do**
2: The data set was randomly divided into ten equal parts;
3: Keep one part as a test data; The remaining nine parts are used as training data;
4: **while** **X** is not empty **do**
5: Train a model based on training data of **X** using SVM;
6: Calculate the prediction accuracy of the model using the test data;
7: Obtain the weight of each gene produced from SVM;
8: Remove *s* least weighted genes and update **X**;
9: **end while**
10: Obtain the gene subset *G*_1_ with the highest prediction accuracy;
11: **while** **X** is not empty **do**
12: Train a model based on training data of **X** using RF;
13: Calculate the prediction accuracy of the model using the test data;
14: Obtain the importance of each gene produced from RF;
15: Remove *s* least weighted genes and update **X**;
16: **end while**
17: Obtain the gene subset *G*_2_ with the highest prediction accuracy;
18: Count the votes for all the genes contained in both *G*_1_ and *G*_2_;
19: **end for**
20: Rank genes based on votes and obtain *G*_*R*_.

#### 2.3.2. Determination of the Optimal Gene Subset

In our study, we generated different gene subsets, gathered all the genes selected through different gene selection algorithms, and chose an optimal gene subset according to the votes for each gene. We assume that *G*_*final*_ is the gene subset eventually selected, and there are *p* genes in *G*_*final*_. According to the votes we obtained for each gene, *G*_*final*_ is acquired as follows:

(2) $$\begin{array}{r} G_{final}=G_{r}:\left\{G_{r1},\cdots \;,G_{rl}\right\}|max\left(Acc\left(G_{r},t_{0}\right)\right),\\ t_{f}>t_{0},t_{f}\in \left[1,10N\right],t_{0}\in \left[0,10N-1\right]. \end{array}$$

where *G*_*r*_ is the top ranked *l* genes of *G*_*R*_; Each of these *l* genes present vote value *t*_*f*_ larger than a threshold *t*_0_. *Acc()* means the accuracy values of *G*_*r*_. Assuming we integrated *N* gene selection algorithms, and thus we would have *N* ten-fold cross validation, respectively. Since all the selected subsets would be put into the voting pool, it made that the number of votes for each gene ranged from 0 to 10 × *N*. Therefore, the *t*_*f*_ ranges from 1 to 10 × *N* and the threshold *t*_0_ ranged from 0 to 10 × *N* − 1. Each time, we selected genes with *t*_*f*_ larger than *t*_0_ and tested the performance for the selected genes. As we set various *t*_0_ values and each *t*_0_ corresponded to a gene subset with *l* genes, the performance using this subset could be calculated. Thus, we obtained a list of accuracy values corresponding to each *t*_0_. Then the subset with the highest accuracy was selected as the final gene subset.

### 2.4. Performance Measurements

Classification sensitivity, specificity and accuracy are important indicators for performance evaluation, which are widely used in diverse applications (Zeng et al., 2017b; Wei et al., 2018b, 2019c; Jin et al., 2019; Zhang et al., 2019b). In this study, we used these three measurements to estimate the performance of the gene subset. They are formulated as follows:

(3) $$\begin{array}{l} \begin{array}{l} \text{Sensitivity(Sen)}=\frac{\text{TP}}{\text{TP}+\text{FN}}\times 100\%,\\ \text{Specificity(Spe)}=\frac{\text{TN}}{\text{TN}+\text{FP}}\times 100\%,\\ \text{Accuracy(Acc)}=\frac{\text{TP}+\text{TN}}{\text{TP}+\text{FP}+\text{FN}+\text{TN}}\times 100\%. \end{array} \end{array}$$

The receive operating characteristic (ROC) curves as well as the area under the ROC, named AUC, were also implemented to measure the performance.

## 3. Experimental Results

### 3.1. Performance Using Different Voting Threshold

Theoretically, the proposed WERFE can ensemble any number of gene selection algorithms. Here in order to made the calculation efficient, we integrated two of the most popular wrapper gene selection algorithms, the RFRFE and SVMRFE, and performed the ten-fold cross validation to pick the most informative genes. In each fold, using the same data splitting strategy, RFRFE and SVMRFE selected their gene subsets respectively. Then we obtained 20 gene subsets considering the ten-fold cross validation. These gene subsets were gathered and put into the voting pool. Based on votes of each gene, we obtained gene rank *G*_*R*_, which is in descending order. Then we re-generated gene subsets by setting different threshold *t*_0_. We evaluated the classification performance of each new gene subset and made the final decision. Here we used RF and SVM as the classifier respectively after obtaining the final gene subset. We used RatinvitroH to validate the WERFE as it is high in dimension. Table 2 shows part of the intermediate outcome of applying WERFE method to RatinvitroH data set. Here as the vote of each gene ranges from 1 to 20, we set the threshold *t*_0_ from 0 to 19.

**Table 2:** Voting and predicted results on RatinvitroH data set using WERFE.

***t*_f_**	***t*_f_**	**GN^a^**	**Acc.RF^b^**	**Sen.RF**	**Spe.RF**	**Acc.SVM**	**Sen.SVM**	**Spe.SVM**
19	20	0	–	–	–	–	–	–
18	19, 20	2	75.79	74.58	56.19	60.45	100	0
17	18–20	17	77.30	81.10	47.26	57.80	95.42	3.33
16	17–20	685	77.15	81.46	48.10	76.67	90.69	60.48
15	16–20	1,092	77.43	85.82	53.10	75.00	82.27	69.76
14	15–20	6,142	75.70	80.17	43.10	65.53	69.57	65.48
0	1–20	31,042	76.84	81.74	66.62	60.23	49.52	50.71

a *GN, gene number*.

b *Acc.RF, Acc using RF as classifier. Other abbreviations in the first row mean in the same way*.

From Table 2, it shows that no gene has 20 votes. It can also be seen that RF performs significantly better than SVM. Two genes obtain 19 votes, and the classification using gene subset composed of these two genes has reached 75.95% of accuracy, 74.58% of sensitivity, and 56.19% of specificity, based on RF. With the increase of the number of genes in the gene subset, the classification accuracy ranges from 75.70 to 77.43%, sensitivity ranges from 74.58 to 85.82%, and specificity ranges from 43.10 to 66.62%, using RF evaluation method. The accuracy achieves the highest when the *t*_0_ is set to 15. However, a huge number of genes are obtained, which makes the computation slow down. In order to balance the gene number and the accuracy, we selected 17 genes as the final gene subset when *t*_0_ equals to 17 and *t*_*f*_ ranges from 18 to 20, and obtained an accuracy of 77.30%, sensitivity of 81.10%, and specificity of 47.26%. That means we can obtain a relatively high classification result with a small number of genes.

### 3.2. Comparison and Analysis With Non-ensemble Algorithms

In theory, our ensemble strategy assumes that integrating more gene selection algorithms is able to give better performance, yet will lead to large calculation cost. Here we only integrated two wrapper algorithms, RFRFE and SVMRFE in the proposed WERFE. We compared WERFE with RFRFE and SVMRFE, respectively and show the results in Tables 3, 4. The comparison was made based on the five data sets.

**Table 3:** Comparison with RFRFE.

**Dataset**	**WERFE**				**RFRFE**			
	**GN^a^**	**Acc**	**Sen**	**Spe**	**GN^a^**	**Acc**	**Sen**	**Spe**
RatinvitroH	17	77.30	81.10	47.26	11	72.27	68.71	34.95
Nki70	5	82.27	49.75	86.13	43	80.15	35.36	83.92
ZQ_188D	1	93.81	98.43	100.00	41	95.80	17.29	99.98
Prostate	4	98.00	95.00	100.00	3	95.31	90.00	100.00
Regicor	4	76.54	65.34	62.71	5	77.76	68.95	64.70

a *GN, gene number*.

**Table 4:** Comparison with SVMRFE.

**Dataset**	**WERFE**				**SVMRFE**			
	**GN^a^**	**Acc**	**Sen**	**Spe**	**GN^a^**	**Acc**	**Sen**	**Spe**
RatinvitroH	17	77.30	81.10	47.26	51	70.30	80.86	53.79
Nki70	5	82.27	49.75	86.13	25	77.10	57.42	88.17
ZQ_188D	1	93.81	98.43	100.00	1	93.81	0	100.00
Prostate	4	98.00	95.00	100.00	42	98.00	96.67	100.00
Regicor	4	76.54	65.34	62.71	3	65.33	62.21	72.24

a *GN, gene number*.

In Table 3, for RatinvitroH, Nki70 and Prostate, it can be clearly seen that the classification accuracy of WERFE is similar or higher than the RFRFE method and the gene subset number is similar or less; while for ZQ_188D and Regicor, although the performance is slightly lower, the gene number is also smaller. The overall performance of WERFE is better than the RFRFE.

From Table 4, we can find that the WERFE performs better on all the five data set than SVMRFE. The accuracy is higher or similar and gene number is smaller or similar.

Comparing across tables, we find WERFE outperforms the other two methods. For example, Nki70's classification accuracy reaches 82.27% using WERFE algorithm. While using RFRFE, the accuracy is 80.15% (Table 3) and using SVMRFE, the classification accuracy is 77.10% (Table 4). The number of selected genes is 5, 43, and 25, respectively. WERFE achieves the highest accuracy using the least number of genes. It is obvious to see the similar trend for the other data sets. Even the accuracy is lower using WERFE, e.g., for data ZD_188D, the accuracy is 2% lower, the much smaller number of gene subset can compensate the slight decrease of accuracy.

**Figure 2:**
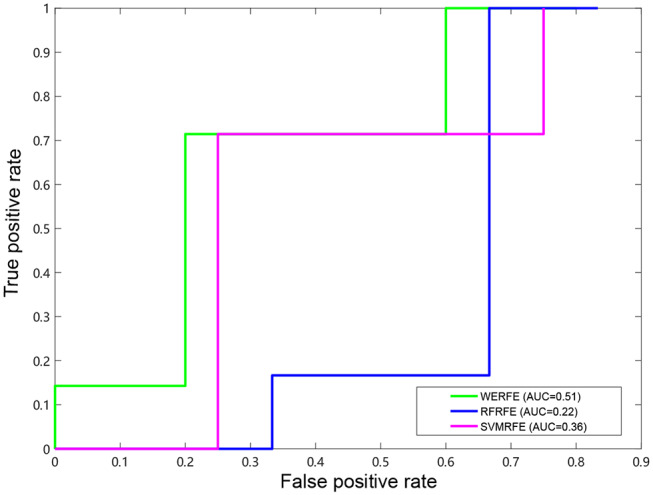
ROC curve on RatinvitroH dataset.

**Figure 3:**
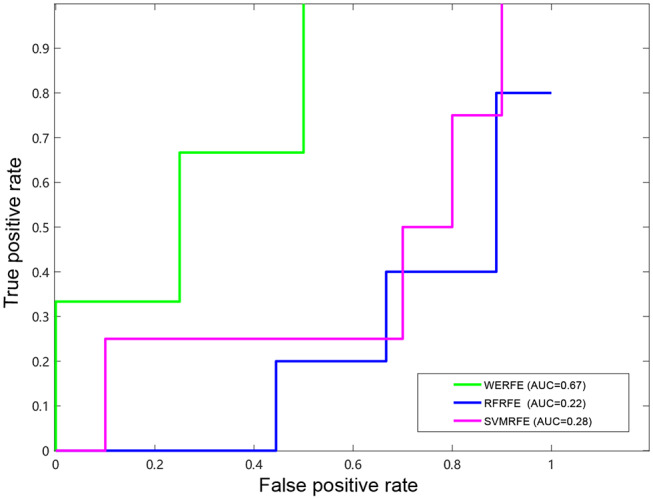
ROC curve on Nki70 dataset.

Figures 2, 3 show the ROC curves of the three methods on RatinvtroH and Nki70 data set. WERFE stays on the top left of RFRFE and SVMRFE, which shows it performs better on RatinvtroH and Nki70 data sets than the other two methods.

### 3.3. Validation Using Other Classifiers

We have shown the results of WERFE using both RF and SVM as the classifiers in section 3.1. Besides classification, RF and SVM also provide gene ranking criteria for WERFE. In order to provide a fair evaluation of WERFE, we used another algorithm, LightGBM algorithm to classify the five data sets and we compared the results with or without WERFE gene selection. LightGBM, a gradient Boosting framework proposed in recent years (Ke et al., 2017), is a distributed and efficient machine learning algorithm based on Gradient Boosting Decision Tree (GBDT) with two key techniques, Gradient-based One-Side Sampling (GOSS), and Exclusive Feature Bundling (EFB). It has been used in gene studies and shown impressive performance (Su et al., 2019e). We show the results using lightGBM with WERFE and lighGBM without WERFE in Table 5.

**Table 5:** Performance between lightGBM with WERFE and without WERFE.

**Dataset**	**With WERFE**				**Without WERFE**			
	**GN^a^**	**Acc**	**Sen**	**Spe**	**GN^a^**	**Acc**	**Sen**	**Spe**
RatinvitroH	17	77.30	81.10	47.26	31042	59.13	73.90	36.93
Nki70	5	82.27	49.75	86.13	70	63.60	31.25	80.00
ZQ_188D	1	93.81	98.43	100.00	188	96.80	61.50	98.90
Prostate	4	98.00	95.00	100.00	100	89.80	88.00	91.70
Regicor	4	76.54	65.34	62.71	22	59.90	64.00	55.70

a *GN, gene number*.

Table 5 shows that, with the exception of the ZQ_188D data set, the classification accuracy and sensitivity of lightGBM plus WERFE is much higher than that of using LightGBM alone. And the WERFE greatly reduces the gene number. This shows that WERFE algorithm performs well in gene selection of most data sets and achieves the purpose of using fewer genes to reach higher classification accuracy.

### 3.4. Comparison With Other Gene Selection Algorithms

We also compared the WERFE with some widely used gene selection approaches including Nie et al.'s method (Nie et al., 2010), Fisher score-based approach and ReliefF approach (Kononenko et al., 1997). We denoted them with FSNM, Fisher, and ReliefF, respectively. These three gene algorithms were conducted combining an incremental search method (ISM). Firstly, the genes were ranked (descending order) using FSNM, Fisher score, and ReliefF, respectively. Then according to the rank, we assumed the basic gene subset include the top ranked θ genes. Next, by adding step size genes each time on top of the basic gene subset, we constructed a group of gene subsets. In order to be consistent with the evaluation method of WERFE algorithm, we also used RF and SVM as the classification methods, and took the subset with the highest accuracy as the result of gene selection. In our study, we set θ to 10 and the step size to 10. The results are shown in Tables 6, 7 for data RatinvitroH and Nki70, respectively.

**Table 6:** Comparison with other gene selection algorithms on RatinvitroH.

**Algorithms**	**RF**				**SVM**			
	**GN^a^**	**Acc**	**Sen**	**Spe**	**GN^a^**	**Acc**	**Sen**	**Spe**
WERFE	17	77.30	81.10	47.26	685	76.67	90.69	60.48
FSNM	60	77.50	83.65	43.52	100	74.85	83.95	60.02
Fisher	20	73.39	69.60	34.02	10	59.85	93.02	14.83
ReliefF	40	73.21	74.60	40.45	80	62.20	97.46	8.17

a *GN, gene number*.

**Table 7:** Comparison with other gene selection algorithms on Nki70.

**Algorithms**	**RF**				**SVM**			
	**GN^a^**	**Acc**	**Sen**	**Spe**	**GN^a^**	**Acc**	**Sen**	**Spe**
WERFE	5	82.27	49.75	86.13	5	72.33	33.00	92.17
FSNM	63	80.85	22.93	88.06	28	81.33	61.79	90.86
Fisher	35	81.46	35.33	92.94	35	74.24	46.12	89.14
ReliefF	21	80.31	39.36	82.11	35	75.76	50.62	87.86

a *GN, gene number*.

Table 6 shows that, in the RF column, FSNM algorithm uses the gene subset composed of 60 genes to obtain the classification accuracy of 77.50%, which is the highest among the four algorithms, and the classification accuracy obtained by WERFE algorithm by using the gene subset composed of 17 genes is 77.30%. Through the comparison of FSNM and WERFE, we find that, although the classification accuracy is similar, the number of genes selected by WERFE algorithm is 20, while the number of genes selected by FSNM is 60, which is 40 more than that of WERFE. Therefore, it is reasonable to choose the WERFE in real applications considering both performance and computation consumption. In the SVM column, the WERFE selects more genes than FSNM but achieved an increase of 2% of accuracy.

Similarly, we applied these gene selection algorithms on the Nki70 dataset. Table 7 shows a comparison of the results of these methods. For the RF column, it is easy to find that WERFE method has the highest classification accuracy 82.27%, when 5 genes were selected as the gene subset. But in the SVM column the WERFE has the worst performance. This indicates that it is better to combine WERFE with RF to perform the gene selection and classification.

## 4. Conclusion

A good gene selection can improve the performance of the classification and play an important role in further analysis. It should take both gene number and classification accuracy into account. In this paper, we proposed an ensemble gene selection algorithm, WERFE, which belongs to a wrapper method within a RFE framework, and conducts the gene selection combining cross validation. The WERFE takes good advantages of multiple gene selection algorithms. Through evaluating each gene with different gene selection algorithms, a small set of genes are selected and the classification accuracy is also improved.

It is expected that better performance can be achieved if integrating more gene selection algorithms. Our study integrates two gene selection algorithms in order to reduce the computation cost. Some of our operations are inspired by the non-ensemble embedded algorithm that we proposed in previous studies (Chen et al., 2018). For instance, we also completed the integration of the algorithm within ten-fold cross-validation. In each fold, under the same training set and test set, different gene selection algorithms were used to obtain the optimal gene subsets, respectively. Then we put the genes contained in each subset of each fold into a voting pool to obtain the votes for each gene. The number of votes of each gene in the voting pool is an important indicator for us to evaluate the gene's importance and based on the votes, we obtained a gene ranking. We constructed new gene subsets according to the ranking and a pre-set threshold was set. Eventually each gene subset was evaluated and a final gene subset was selected.

We used five data sets (RatinvitroH, Nki70, ZQ_180D, Prostate, and Regicor) to validate the proposed method. In order to verify the effectiveness of the gene selection algorithm, we designed three groups of comparative experiments. Firstly, we chose two wrapper algorithms, which are also the two basic algorithms integrated into our proposed algorithm, to compare with the WERFE. The results show that the proposed method outperforms the other two wrapper algorithms. Secondly, we used another classification algorithm, lightGBM, to evaluate the proposed method. We compared the performance between methods using WERFE and not using WERFE. And the results show that lightGBM performs better when using WERFE. Finally, we compared the WERFE with three other gene selection algorithms. It shows from the results that WERFE is best in both improving classification accuracy and reducing gene number. However, there are some limitations of the proposed method. For instance, this method needs to consume more computing resources if more gene selection algorithms are integrated. When the number of genes is large, the operation time will be relatively long.

In the future, we will test this algorithm on more types of data sets to further improve the algorithm. At the same time, we will also try to integrate more gene selection methods, aiming to evaluate the importance of genes in a more objective way, and meanwhile reduce the calculation time. We target to solve this through deep learning method.

## Data Availability Statement

Publicly available datasets were analyzed in this study. This data can be found here: http://toxico.nibio.go.jp/english/index.html; http://crdd.osdd.net/raghava/cppsite/.

## Author Contributions

RS conceived and designed the experiments and revised the manuscript. QC collected the data, performed the analysis, and wrote the paper. ZM contributed the analysis tools and participated in revising the manuscript.

## Conflict of Interest

The authors declare that the research was conducted in the absence of any commercial or financial relationships that could be construed as a potential conflict of interest.

## References

[B1] Chen Q. Meng Z. Liu X. Jin Q. Su R. (2018). Decision variants for the automatic determination of optimal feature subset in RF-RFE. Genes 9:301. 10.3390/genes9060301 29914084 PMC6027449

[B2] Cortes C. Vapnik V. (1995). Support-vector networks. Mach. Learn. 20, 273–297. 10.1007/BF00994018

[B3] Dash M. Liu H. (1997). Feature selection for classification. Intell. Data Anal. 1, 131–156. 10.3233/IDA-1997-1302

[B4] Duval B. Hao J. K. Hernandez J. C. H. (2009). “A memetic algorithm for gene selection and molecular classification of cancer,” in Genetic & Evolutionary Computation Conference (Montreal, CA), 201–208. 10.1145/1569901.1569930

[B5] Fajila M. N. F. (2019). Gene subset selection for leukemia classification using microarray data. Curr. Bioinformatics 14, 353–358. 10.2174/1574893613666181031141717

[B6] Gautam A. Singh H. Tyagi A. Chaudhary K. Kumar R. Kapoor P.. (2012). CPPsite: a curated database of cell penetrating peptides. Database 2012:bas015. 10.1093/database/bas015 22403286 PMC3296953

[B7] Gong Y. Niu Y. Zhang W. Li X. (2019). A network embedding-based multiple information integration method for the MiRNA-disease association prediction. BMC Bioinformatics 20:468. 10.1186/s12859-019-3063-3 31510919 PMC6740005

[B8] Gu Q. Li Z. Han J. (2011). “Generalized fisher score for feature selection,” in Twenty-seventh Conference on Uncertainty in Artificial Intelligence (Barcelona), 266–273.

[B9] Guyon I. Weston J. Barnhill S. Vapnik V. (2002). Gene selection for cancer classification using support vector machines. Mach. Learn. 46, 389–422. 10.1023/A:1012487302797

[B10] Hernandez J. C. H. Hao J. K. (2007). “A genetic embedded approach for gene selection and classification of microarray data,” in European Conference on Evolutionary Computation (Valencia), 90–101. 10.1007/978-3-540-71783-6_9

[B11] Ho T. K. (1995). “Random decision forests,” in International Conference on Document Analysis & Recognition (Montreal, CA), 278–282.

[B12] Jin Q. Meng Z. Pham T. D. Chen Q. Wei L. Su R. (2019). DUNet: A deformable network for retinal vessel segmentation. Knowl. Based Syst. 178, 149–162. 10.1016/j.knosys.2019.04.025

[B13] John G. Kohavi R. Pfleger K. (1994). “Irrelevant features and the subset selection problem,” in Machine Learning Proceedings (New Brunswick; New Jersey, NJ), 121–129. 10.1016/B978-1-55860-335-6.50023-4

[B14] Ke G. Meng Q. Finley T. Wang T. Chen W. Ma W. (2017). “LightGBM: a highly efficient gradient boosting decision tree,” in 31st Conference on Neural Information Processing Systems (Long Beach, CA), 3149–3157.

[B15] Kira K. Rendell L. A. (1992). “The feature selection problem: traditional methods and a new algorithm,” in Tenth National Conference on Artificial Intelligence (San Jose, CA), 129–134.

[B16] Koller D. Sahami M. (1996). “Toward optimal feature selection,” in Thirteenth International Conference on International Conference on Machine Learning (Bari), 284–292.

[B17] Kononenko I. Simec E. Robnik-Sikonja M. (1997). Overcoming the myopia of inductive learning algorithms with RELIEFF. Appl. Intell. 7, 39–55. 10.1023/A:1008280620621

[B18] Lambrou G. I. Sdraka M. Koutsouris D. (2019). The “gene cube”: A novel approach to three-dimensional clustering of gene expression data. Curr. Bioinformatics 14, 721–727. 10.2174/1574893614666190116170406

[B19] Li Yeh C. Cheng-Huei Y. Cheng Hong Y. (2009). Tabu search and binary particle swarm optimization for feature selection using microarray data. J. Comput. Biol. J. Comput. Mol. Cell Biol. 16, 1689–1703. 10.1089/cmb.2007.0211 20047491

[B20] Liu Y. Zeng X. He Z. Zou Q. (2017). Inferring MicroRNA-disease associations by random walk on a heterogeneous network with multiple data sources. IEEE/ACM Trans. Comput. Biol. Bioinformatics 14, 905–915. 10.1109/TCBB.2016.2550432 27076459

[B21] Lv Z. Jin S. Ding H. Zou Q. (2019). A random forest sub-golgi protein classifier optimized via dipeptide and amino acid composition features. Front. Bioeng. Biotechnol. 7:215. 10.3389/fbioe.2019.00215 31552241 PMC6737778

[B22] Nie F. Huang H. Cai X. Ding C. (2010). “Efficient and robust feature selection via joint ℓ_21-norms minimization,” in Proceedings of the 23rd International Conference on Neural Information Processing Systems, Vol. 2 (Kyoto), 1813–1821.

[B23] Otava M. Shkedy Z. Kasim A. (2014). Prediction of gene expression in human using rat in *vivo* gene expression in Japanese toxicogenomics project. Syst. Biomed. 2, 8–15. 10.4161/sysb.29412

[B24] Peng H. C. Long F. H. Ding C. (2005). Feature selection based on mutual information criteria of max-dependency, max-relevance, and min-redundancy. IEEE Trans. Pattern Anal. Mach. Intell. 27, 1226–1238. 10.1109/TPAMI.2005.159 16119262

[B25] Phuong T. M. Lin Z. Altman R. B. (2005). “Choosing SNPs using feature selection,” in Computational Systems Bioinformatics Conference (Stanford, CA), 301–309. 10.1109/CSB.2005.22 16447987

[B26] Piyush A. Sherry B. Sadullah U. S. Sandeep S. Kumardeep C. S. Ankur G. (2015). CPPsite 2.0: a repository of experimentally validated cell-penetrating peptides. Nucleic Acids Res. 44, D1098–D1103. 10.1093/nar/gkv1266 26586798 PMC4702894

[B27] Robnik-Sikonja M. Kononenko I. (1997). “An adaptation of relief for attribute estimation in regression,” in Fourteenth International Conference on Machine Learning (Nashville, TN), 296–304.

[B28] Roffo G. Melzi S. Cristani M. (2016). “Infinite feature selection,” in IEEE International Conference on Computer Vision (Santiago), 4202–4210. 10.1109/ICCV.2015.478

[B29] Shah S. C. Kusiak A. (2004). Data mining and genetic algorithm based gene/SNP selection. Artif. Intell. Med. 31, 183–196. 10.1016/j.artmed.2004.04.002 15302085

[B30] Shahjaman M. Kumar N. Mollah N. H. (2019). Performance improvement of gene selection methods using outlier modification rule. Curr. Bioinformatics 14, 491–503. 10.2174/1574893614666181126110008

[B31] Su R. Liu T. Sun C. Jin Q. Jennane R. Wei L. (2019a). Fusing convolutional neural network features with hand-crafted features for osteoporosis diagnoses. Neurocomputing. 385, 300–309. 10.1016/j.neucom.2019.12.083

[B32] Su R. Liu X. Wei L. (2019b). MinE-RFE: determine the optimal subset from RFE by minimizing the subset-accuracy-defined energy. Brief. Bioinformatics. 10.1093/bib/bbz021 30860571

[B33] Su R. Liu X. Wei L. Zou Q. (2019c). Deep-Resp-Forest: A deep forest model to predict anti-cancer drug response. Methods 166, 91–102. 10.1016/j.ymeth.2019.02.009 30772464

[B34] Su R. Liu X. Xiao G. Wei L. (2019d). Meta-GDBP: a high-level stacked regression model to improve anti-cancer drug response prediction. Brief. Bioinformatics. 10.1093/bib/bbz022 30868164

[B35] Su R. Wu H. Liu X. Wei L. (2019e). Predicting drug-induced hepatotoxicity based on biological feature maps and diverse classification strategies. Brief. Bioinformatics. 10.1093/bib/bbz165 31838506

[B36] Su R. Wu H. Xu B. Liu X. Wei L. (2018). Developing a multi-dose computational model for drug-induced hepatotoxicity prediction based on toxicogenomics data. IEEE/ACM Trans. Comput. Biol. Bioinformatics 16, 1231–1239. 10.1109/TCBB.2018.2858756 30040651

[B37] Su R. Zhang J. Liu X. Wei L. (2019f). Identification of expression signatures for Non-Small-Cell Lung Carcinoma subtype classification. Bioinformatics. 36, 339–346. 10.1093/bioinformatics/btz557 31297509

[B38] Subirana I. Sanz H. Vila J. (2014). Building bivariate tables: the comparegroups package for R. J. Stat. Softw. 57, 1–16. 10.18637/jss.v057.i1225400517

[B39] Torrente A. López-Pintado S. Romo J. (2013). DepthTools: an R package for a robust analysis of gene expression data. BMC Bioinformatics 14:237. 10.1186/1471-2105-14-237 23885712 PMC3750619

[B40] Wang B. Lu K. Long H. Zhou Y. Zheng C.-H. Zhang J. (2018). Early stage identification of Alzheimer's disease using a two-stage ensemble classifier. Curr. Bioinformatics 13, 529–535. 10.2174/1574893613666180328093114

[B41] Wang Y. Shi F. Cao L. Dey N. Wu Q. Ashour A. S. (2019). Morphological segmentation analysis and texture-based support vector machines classification on mice liver fibrosis microscopic images. Curr. Bioinformatics 14, 282–294. 10.2174/1574893614666190304125221

[B42] Wei L. Chen H. Su R. (2018a). M6APred-EL: a sequence-based predictor for identifying n6-methyladenosine sites using ensemble learning. Mol. Ther. Nucleic Acids 12, 635–644. 10.1016/j.omtn.2018.07.004 30081234 PMC6082921

[B43] Wei L. Ding Y. Su R. Tang J. Zou Q. (2018b). Prediction of human protein subcellular localization using deep learning. J. Parallel Distrib. Comput. 117, 212–217. 10.1016/j.jpdc.2017.08.009

[B44] Wei L. Su R. Wang B. Li X. Zou Q. Gao X. (2019a). Integration of deep feature representations and handcrafted features to improve the prediction of N6-methyladenosine sites. Neurocomputing 324, 3–9. 10.1016/j.neucom.2018.04.082

[B45] Wei L. Wan S. Guo J. Wong K. K. (2017a). A novel hierarchical selective ensemble classifier with bioinformatics application. Artif. Intell. Med. 83, 82–90. 10.1016/j.artmed.2017.02.005 28245947

[B46] Wei L. Xing P. Shi G. Ji Z. L. Zou Q. (2019b). Fast prediction of protein methylation sites using a sequence-based feature selection technique. IEEE/ACM Trans. Comput. Biol. Bioinformatics 16, 1264–1273. 10.1109/TCBB.2017.2670558 28222000

[B47] Wei L. Xing P. Su R. Shi G. Ma Z. S. Zou Q. (2019c). CPPred-RF: a sequence-based predictor for identifying cell-penetrating peptides and their uptake efficiency. J. Proteome Res. 16, 2044–2053. 10.1021/acs.jproteome.7b00019 28436664

[B48] Wei L. Xing P. Zeng J. Chen J. Su R. Guo F. (2017b). Improved prediction of protein-protein interactions using novel negative samples, features, and an ensemble classifier. Artif. Intell. Med. 83, 67–74. 10.1016/j.artmed.2017.03.001 28320624

[B49] Yoshinobu I. Noriyuki N. Tomoya Y. Atsushi O. Yasuo O. Tetsuro U. (2015). Open TG-GATEs: a large-scale toxicogenomics database. Nucleic Acids Res. 43:D921 10.1093/nar/gku955 25313160 PMC4384023

[B50] Zeng X. Liao Y. Liu Y. Zou Q. (2017a). Prediction and validation of disease genes using HeteSim scores. IEEE/ACM Trans. Comput. Biol. Bioinformatics 14, 687–695. 10.1109/TCBB.2016.2520947 26890920

[B51] Zeng X. Lin W. Guo M. Zou Q. (2017b). A comprehensive overview and evaluation of circular RNA detection tools. PLoS Comput. Biol. 13:e1005420. 10.1371/journal.pcbi.1005420 28594838 PMC5466358

[B52] Zeng X. Liu L. Lü L. Zou Q. (2018). Prediction of potential disease-associated microRNAs using structural perturbation method. Bioinformatics 34, 2425–2432. 10.1093/bioinformatics/bty112 29490018

[B53] Zhang N. Sa Y. Guo Y. Lin W. Wang P. Feng Y. (2018). Discriminating ramos and jurkat cells with image textures from diffraction imaging flow cytometry based on a support vector machine. Curr. Bioinformatics 13, 50–56. 10.2174/1574893611666160608102537

[B54] Zhang W. Jing K. Huang F. Chen Y. Li B. Li J. (2019a). SFLLN: A sparse feature learning ensemble method with linear neighborhood regularization for predicting drug-drug interactions. Inform. Sci. 497, 189–201. 10.1016/j.ins.2019.05.017

[B55] Zhang W. Li Z. Guo W. Yang W. Huang F. (2019b). A fast linear neighborhood similarity-based network link inference method to predict microRNA-disease associations. IEEE/ACM Trans. Comput. Biol. Bioinformatics 1–1. 10.1109/TCBB.2019.2931546 31369383

[B56] Zhang W. Yue X. Tang G. Wu W. Huang F. Zhang X. (2018). SFPEL-LPI: Sequence-based feature projection ensemble learning for predicting lncRNA-protein interactions. PLoS Comput. Biol. 14:e1006616. 10.1371/journal.pcbi.1006616 30533006 PMC6331124

[B57] Zhang Y. Dong Z. Phillips P. Wang S. Ji G. Yang J.. (2015). Detection of subjects and brain regions related to Alzheimer's disease using 3D MRI scans based on eigenbrain and machine learning. Front. Comput. Neurosci. 9:66. 10.3389/fncom.2015.00066 26082713 PMC4451357

[B58] Zhang Y. Wang S. Phillips P. Ji G. (2014). Binary PSO with mutation operator for feature selection using decision tree applied to spam detection. Knowl. Based Syst. 64, 22–31. 10.1016/j.knosys.2014.03.015

